# Supplementary material for: Sex-differentiated placental methylation and gene expression regulation has implications for neonatal traits and adult diseases
Source: Nat Commun. 2025 May 1;16:4004. doi: 10.1038/s41467-025-58128-3 (PMC12045980; doi:10.1038/s41467-025-58128-3)

SUPPLEMENTRAY INFORMATION

Sex-differentiated placental methylation and gene expression has implications for neonatal traits and adult diseases

Fasil Tekola-Ayele *et al*

Supplementary Figures

Supplementary Fig. 1. Correlation between methylation principal components (PCs) and placenta cell composition derived in silico using DNA methylation data. methPC denotes methylation PC. nRBC denotes nucleated red blood cells. Numbers inside boxes represent Spearman correlation coefficient. An "X" inside a box indicates that the correlation is not statistically significant based on a two-sided Spearman correlation test using the S statistic. Axes list the variables being tested for correlation. Color of the box indicates the magnitude and direction of the correlation. Correlations were considered significant if P < 0.05. n = 291 placental DNA methylation samples.

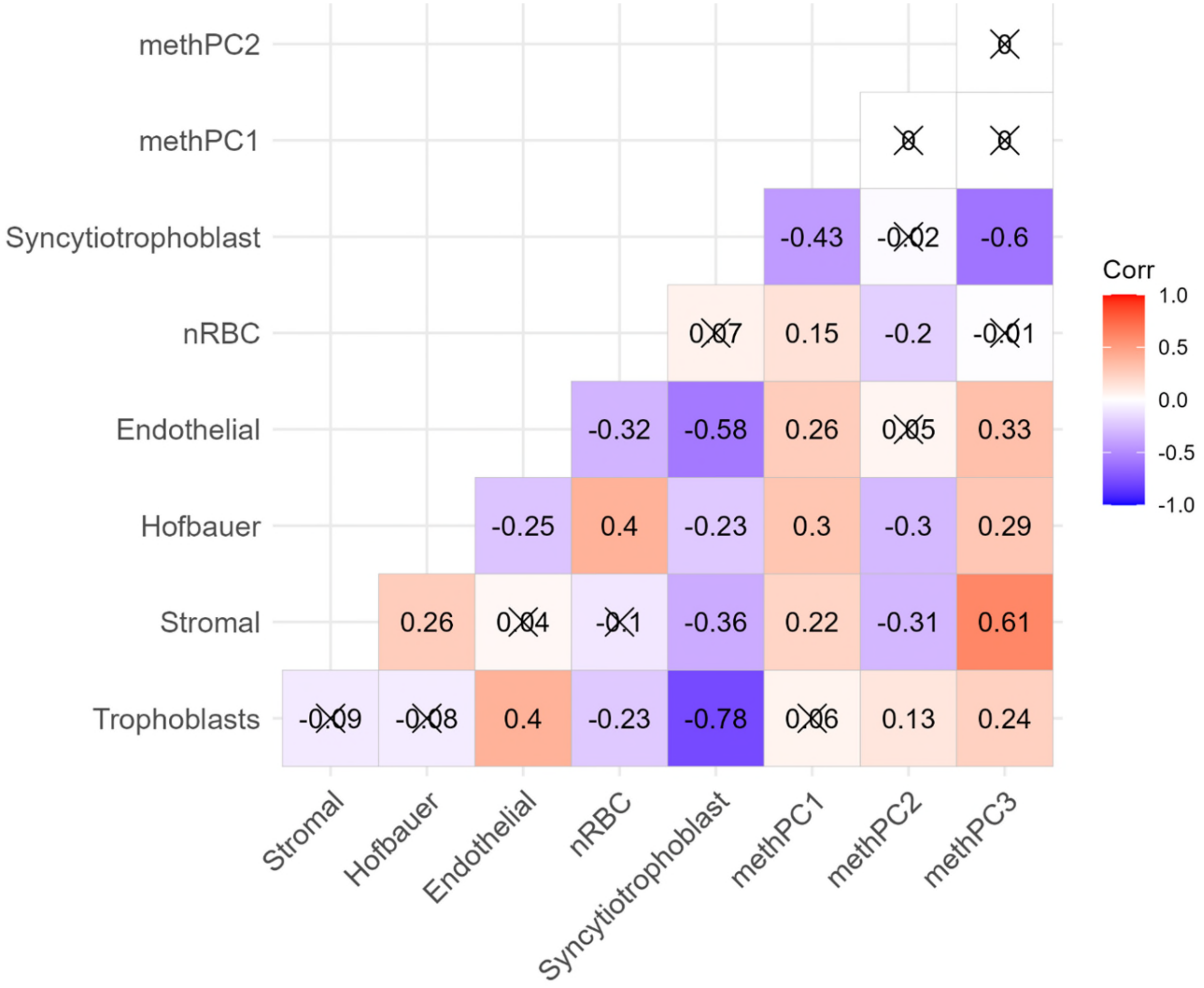

Supplementary Fig. 2. Correlation between RNA principal components (PCs) and placenta cell composition derived in silico using RNA-seq data. Numbers inside boxes represent Spearman correlation coefficient. An “X” inside a box indicates that the correlation is not statistically significant based on a two-sided Spearman correlation test using the S statistic. Axes list the variables being tested for correlation. Color of the box indicates the magnitude and direction of the correlation. Correlations were considered significant if $P < 0.05$. n = 80 placental RNA-seq samples.

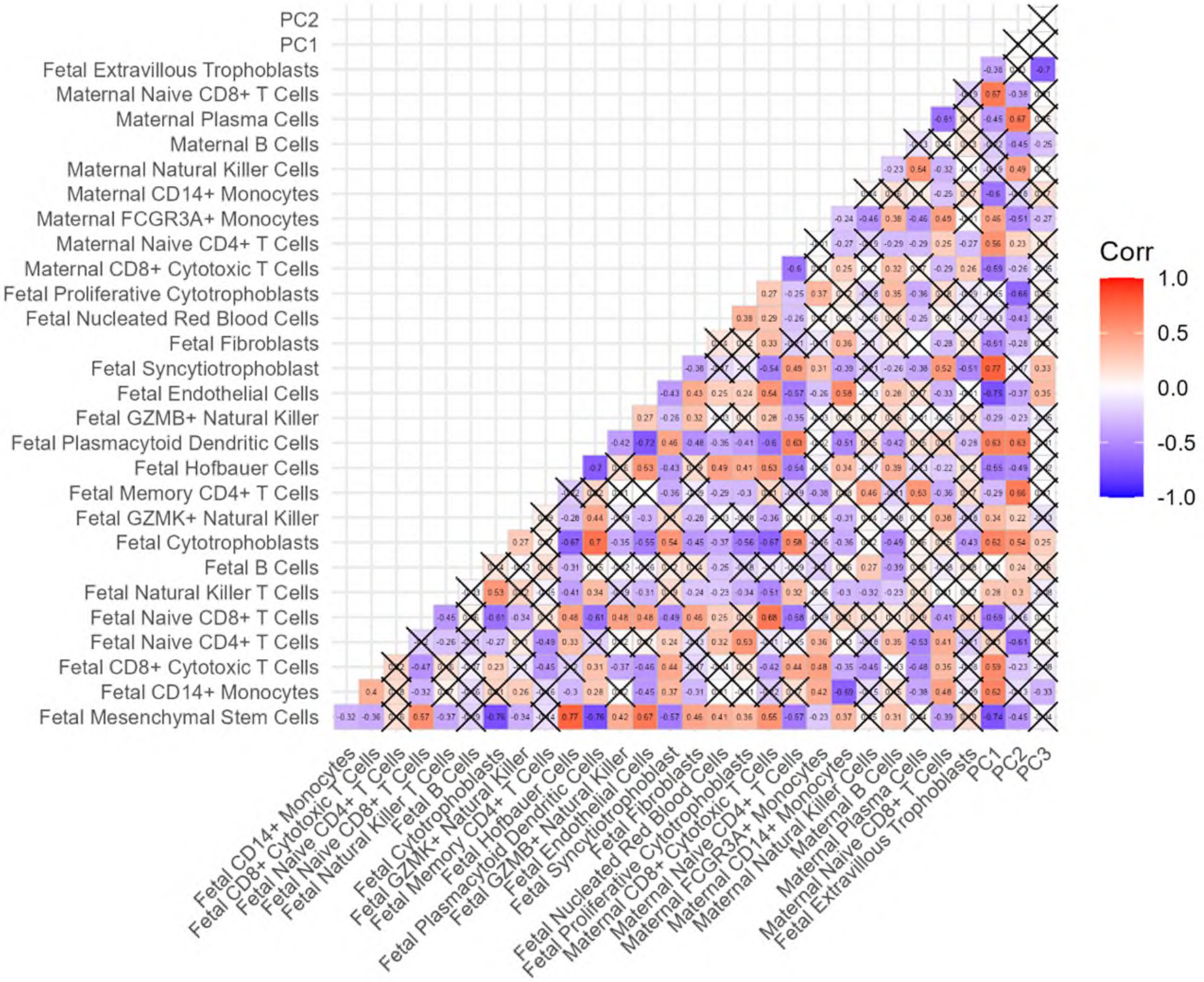

Supplementary Fig. 3. Circos plots of significantly enriched hallmark gene sets for genes near sex-differentially methylated sites. Hallmark gene sets significantly enriched (hypergeometric test FDR *P*<0.05) in males and females. The outer track represents the enriched hallmark gene sets using the FUMA tool, ordered from the most to the least significant in clockwise direction. The middle track represents the enrichment adjusted *P*-value (red is most significant, yellow is least significant). The orange bars in the inner-most track represent the number of genes near the sex-differentially methylated sites that overlapped with genes in the database of each hallmark gene set. The green lines connect enriched hallmark gene sets shared between males (blue) and females (pink), with width proportional to the number of shared genes. H denotes Hallmark. Source data are provided as a Source Data file.

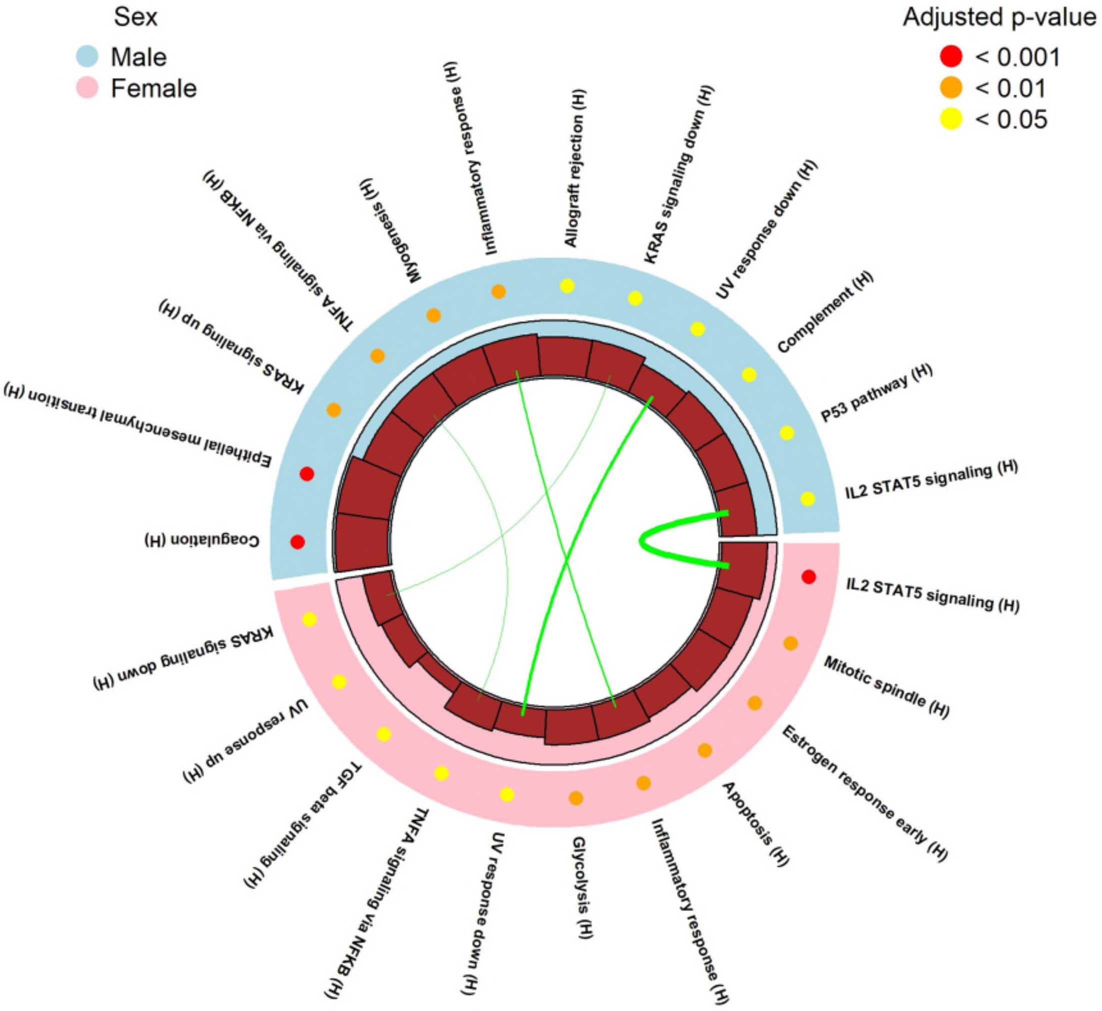

Supplementary Fig. 4. Transcription factor binding site (TFBS) enrichment in genomic regions impacted by placental sex. a Sex-mQTL genetic variants. **b** Sex-mQTL CpG sites. **c** Male-hypermethylated sex-differentially methylated (sex-DM) CpG sites. **d** Female-hypermethylated sex-differentially methylated (sex-DM) CpG sites. The x-axis in **a** represents the transcription factor, sorted by observed/expected matches. Enrichment was tested using hypergeometric test and was considered significant if $P < 0.05$. The color of the points indicate significance in **a** and type of transcription factor targeted in **b-d**.

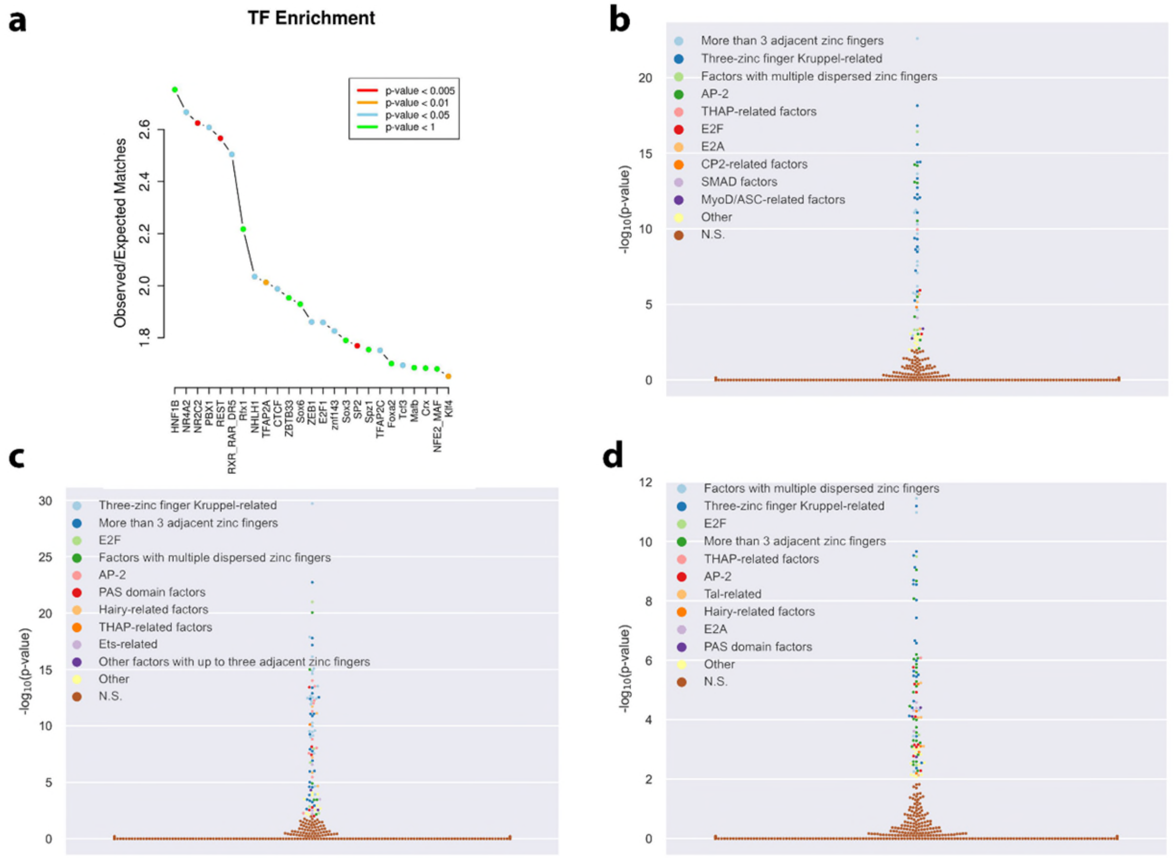

Supplement: Supplementary file 1 — Supplementary Information [file 41467_2025_58128_MOESM1_ESM.pdf]
