# Supplementary material for: Sex-differentiated placental methylation and gene expression regulation has implications for neonatal traits and adult diseases
Source: Nat Commun. 2025 May 1;16:4004. doi: 10.1038/s41467-025-58128-3 (PMC12045980; doi:10.1038/s41467-025-58128-3)
Supplement: Supplementary file 2 — Description of Additional Supplementary Files [file 41467_2025_58128_MOESM2_ESM.pdf]

**Description of Additional Supplementary Files**

File Name: Supplementary Data 1

Description: Characteristics of the study participants in the discovery and replication cohorts. Subsections **a** and **b** contain characteristics corresponding to the discovery and replication cohorts, respectively. Table headers, sections, and column names are bolded for ease of reading. Sex differences were tested using two-sided t-tests for continuous variables and chi-squared tests for categorical variables.

File Name: Supplementary Data 2

Description: DNA methylation CpG sites that are significantly differentially methylated between male and female placenta. P-values < 0.05 are highlighted in green using conditional formatting. Conditional formatting with solid fill blue data bars is applied to logFC and Spearman rho correlation coefficients. Table headers, sections, and column names are bolded for ease of reading. sex-DM was tested using two-sided t-tests with BACON and Benjamini-Hochberg adjustments for multiple testing. Correlation between methylation and birth outcome was tested using two-sided Spearman correlation tests. Correlation between methylation and nearby gene expression was tested using two-sided Spearman correlation tests with Benjamin-Hochberg adjustments for multiple testing.

File Name: Supplementary Data 3

Description: Sex differentially methylated CpG sites significantly correlated with nearby gene expression in placenta. Table headers, sections, and column names are bolded for ease of reading. Correlation between methylation and nearby gene expression was tested using two-sided Spearman correlation tests with Benjamin-Hochberg adjustments for multiple testing.

File Name: Supplementary Data 4

Description: Sex-biased cis-methylation quantitative trait locus association (sex-mQTL) in placenta. P-values < 0.05 are highlighted in green using conditional formatting. Conditional formatting with solid fill blue data bars is applied to sex interaction term beta estimates and Spearman rho correlation coefficients. Table headers, sections, and column names are bolded for ease of reading. sex-mQTL interaction was tested using two-sided t-tests with Benjamin-Hochberg adjustments for multiple testing. Sex-stratified analysis was performed using two-sided t-tests with Benjamin-Hochberg adjustments for multiple testing. Sex difference analysis was performed using two-sided t-tests, assuming unequal variance, with Benjamin-Hochberg adjustments for multiple testing. Correlation between methylation and placental cell type proportion was tested using two-sided Spearman correlation tests with Benjamin-Hochberg adjustments for multiple testing.

File Name: Supplementary Data 5

Description: Sex-mQTL replication results in the Rhode Island Child Health Study (RICHS) cohort. P-values < 0.05 are highlighted in green using conditional formatting. Table headers, sections, and column names

are bolded for ease of reading. sex-mQTL interaction was tested using two-sided t-tests with Benjamin-Hochberg adjustments for multiple testing.

File Name: Supplementary Data 6

Description: Genes that are significantly differentially expressed between male and female placenta. P-values < 0.05 are highlighted in green using conditional formatting. Conditional formatting with solid fill blue data bars is applied to logFC. Table headers, sections, and column names are bolded for ease of reading. sex-DE was tested using two-sided likelihood ratio tests with Benjamin-Hochberg adjustments for multiple testing.

File Name: Supplementary Data 7

Description: Sex-biased cis-expression quantitative trait locus association (sex-eQTL) in placenta. P-values < 0.05 are highlighted in green using conditional formatting. Conditional formatting with solid fill blue data bars is applied to sex interaction term beta estimates and Spearman rho correlation coefficients. eQTLs that have an identified sex effect category are highlighted in yellow using conditional formatting. Table headers, sections, and column names are bolded for ease of reading. sex-eQTL interaction was tested using two-sided t-tests with Benjamin-Hochberg adjustments for multiple testing. Sex-stratified analysis was performed using two-sided t-tests with Benjamin-Hochberg adjustments for multiple testing. Sex difference analysis was performed using two-sided t-tests, assuming unequal variance, with Benjamin-Hochberg adjustments for multiple testing. Correlation between gene expression and placental cell type proportion was tested using two-sided Spearman correlation tests with Benjamin-Hochberg adjustments for multiple testing.

File Name: Supplementary Data 8

Description: Association between placental cell type proportion and sex. Subsections **a** and **b** contain associations corresponding to the cell types derived from DNA methylation and RNA-seq data, respectively. P-values < 0.05 are highlighted in green using conditional formatting. Table headers, sections, and column names are bolded for ease of reading.

File Name: Supplementary Data 9

Description: Overlap between sex-biased loci identified and previous trait associations. Subsection **a** contains EWAS Atlas phenotypes whose associated CpGs overlapped with sex-DM CpGs. Subsection **b** contains GWAS Catalog phenotypes whose associated genes overlapped with genes annotating sex-DM CpGs. Subsection **c** contains identified sex-mQTL CpGs which overlapped with EWAS Catalog CpGs. Subsection **d** contained identified sex-eQTL genes which overlapped with GWAS Catalog genes. Table headers, sections, and column names are bolded for ease of reading. Enrichment in subsections **a** and **b** were tested using hypergeometric tests of enrichment with Benjamin-Hochberg adjustments for multiple testing.

File Name: Supplementary Data 10

Description: Loci with significant colocalization of sex-mQTL or sex-eQTL in placenta and genome-wide association study trait loci. Subsections **a** and **b** contain GWAS colocalization results corresponding to the sex-mQTLs and sex-eQTLs, respectively. Table headers, sections, and column names are bolded for ease of reading.

File Name: Supplementary Data 11

Description: Imprinted genes overlapping with sex differentially methylated (sex-DM) CpG gene annotations. Table headers, sections, and column names are bolded for ease of reading. Every other imprinted gene's rows are heighted in gray for ease of reading.

File Name: Supplementary Data 12

Description: Significantly enriched hallmark gene sets and canonical pathways in sex differentially methylated CpG gene annotations. Subsections **a** and **b** contain hallmark gene sets enriched for genes near sex-DM CpGs hypermethylated in male and female placenta, respectively. Subsections **c** and **d** contain canonical pathways from MsigDB.c2 enriched for genes near sex-DM CpGs hypermethylated in male and female placenta, respectively. Gene sets in **a** and **b** contain hyperlinks to the MsigDB page for the given gene set. Table headers, sections, and column names are bolded for ease of reading. Enrichment was tested using hypergeometric tests of enrichment with Bonferroni adjustments for multiple testing

File Name: Supplementary Data 13

Description: List of genome-wide association studies that met eligibility criteria for colocalization test with sex-eQTL and sex-mQTL loci in placenta. Subsections **a** and **b** contain the GWAS colocalization references used for sex-eQTL and sex-mQTL loci, respectively. Table headers and sections are bolded for ease of reading.
