# Supplementary material for: Sex-differentiated placental methylation and gene expression regulation has implications for neonatal traits and adult diseases
Source: Nat Commun. 2025 May 1;16:4004. doi: 10.1038/s41467-025-58128-3 (PMC12045980; doi:10.1038/s41467-025-58128-3)
Supplement: Supplementary file 16 — Reporting Summary [file 41467_2025_58128_MOESM16_ESM.pdf]

nature portfolio

Corresponding author(s): Fasil Tekola-Ayele

Last updated by author(s): Mar 5, 2025

Reporting Summary

Nature Portfolio wishes to improve the reproducibility of the work that we publish. This form provides structure for consistency and transparency in reporting. For further information on Nature Portfolio policies, see our Editorial Policies and the Editorial Policy Checklist.

Statistics

For all statistical analyses, confirm that the following items are present in the figure legend, table legend, main text, or Methods section.

n/a	Confirmed	
☐	☒	The exact sample size (*n*) for each experimental group/condition, given as a discrete number and unit of measurement
☐	☒	A statement on whether measurements were taken from distinct samples or whether the same sample was measured repeatedly
☐	☒	The statistical test(s) used AND whether they are one- or two-sided<br>*Only common tests should be described solely by name; describe more complex techniques in the Methods section.*
☐	☒	A description of all covariates tested
☐	☒	A description of any assumptions or corrections, such as tests of normality and adjustment for multiple comparisons
☐	☒	A full description of the statistical parameters including central tendency (e.g. means) or other basic estimates (e.g. regression coefficient) AND variation (e.g. standard deviation) or associated estimates of uncertainty (e.g. confidence intervals)
☐	☒	For null hypothesis testing, the test statistic (e.g. *F*, *t*, *r*) with confidence intervals, effect sizes, degrees of freedom and *P* value noted<br>*Give P values as exact values whenever suitable.*
☒	☐	For Bayesian analysis, information on the choice of priors and Markov chain Monte Carlo settings
☒	☐	For hierarchical and complex designs, identification of the appropriate level for tests and full reporting of outcomes
☐	☒	Estimates of effect sizes (e.g. Cohen's *d*, Pearson's *r*), indicating how they were calculated

Our web collection on statistics for biologists contains articles on many of the points above.

Software and code

Policy information about availability of computer code

Data collection	No software was used for data collection.
Data analysis	R/Bioconductor packages used included edgeR, planet, prcomp, limma, LDlinkR, MatrixEQTL, biomaRt, cowplot, tidyverse, ggpubr, ComplexUpset, patchwork, data.table, readxl, gridExtra, circlize, ggcorrplot, rjson, ggfastman, and BACON; Open source software used included CIBERSORTx, TopHat v2.0.4, PLINK v1.9, PEER, VCFtools, BCFtools, bedtools; Open source web platforms used included ezQTL (https://analysistools.cancer.gov/ezqtl/#/home), JASPAR enrichment tool (https://jaspar.genereg.net/), SNP2TFBS (https://epd.expasy.org/snp2tfbs/), FUMA v1.3.6a (https://fuma.ctglab.nl/), Michigan Imputation Server (https://imputationserver.sph.umich.edu/), eFORGE v2.0 (https://eforge.altiusinstitute.org/), LocusZoom (http://locuszoom.org/genform.php?type=yourdata), UCSC Genome Browser (https://genome.ucsc.edu/cgi-bin/hgGateway), Human Protein Atlas (https://www.proteinatlas.org/).

For manuscripts utilizing custom algorithms or software that are central to the research but not yet described in published literature, software must be made available to editors and reviewers. We strongly encourage code deposition in a community repository (e.g. GitHub). See the Nature Portfolio guidelines for submitting code & software for further information.

Data

Policy information about availability of data

All manuscripts must include a data availability statement. This statement should provide the following information, where applicable:

- Accession codes, unique identifiers, or web links for publicly available datasets
- A description of any restrictions on data availability
- For clinical datasets or third party data, please ensure that the statement adheres to our policy

As part of the NICHD Fetal Growth Studies, which is the discovery cohort in the present study, the genotypes, DNA methylation, and gene expression data have been deposited in the dbGaP database under accession code phs001717.v1.p1 (https://www.ncbi.nlm.nih.gov/gap/?term=phs001717.v1.p1). Moreover, as part of the Rhode Island Child Health Study (RICHS), which is the replication cohort in the present study, the genotypes and gene expression data have been deposited in the dbGaP database under accession code phs001586.v1.p1 (https://www.ncbi.nlm.nih.gov/projects/gap/cgi-bin/study.cgi?study_id=phs001586.v1.p1). The following datasets made available by other groups were also used in the present analyses: the 1000 Genomes Reference Panel datasets were accessed at https://www.internationalgenome.org/category/reference/; the human genome reference made accessible by the Genome Reference Consortium was accessed at https://www.ncbi.nlm.nih.gov/assembly/GCF_000001405.13/; genotype imputation platform as well as the Haplotype Reference Consortium reference panel was accessed via the Michigan Imputation Server at https://imputationserver.sph.umich.edu/; and the Human Protein Altas dataset was downloaded at https://www.proteinatlas.org/about/download#protein_atlas_data. Source data are provided with this paper.

Research involving human participants, their data, or biological material

Policy information about studies with human participants or human data. See also policy information about sex, gender (identity/presentation), and sexual orientation and race, ethnicity and racism.

Reporting on sex and gender	As described in the manuscript, we used fetal sex (male, female) collected from study medical charts. The study was designed to develop fetal growth standards, including prediction of small- or large- for gestational age at birth, based on sex-specific population references. Analysis involves an interaction term on sex and genotype, as well as sex-stratified analyses. This analysis involved 152 males and 149 females.
Reporting on race, ethnicity, or other socially relevant groupings	The NICHD Fetal Growth Study was designed to develop race/ethnic standards for fetal growth, hence recruitment was aimed at statistically powering the number of study participants from four race/ethnic groups. Race/ethnicity was self-reported. In the analyses, gestational age at delivery, race/ethnicity (to account for the race/ethnicity-based design), technical covariates (e.g., methylation sample plate and methylation PCs for EWAS; PEER factors for eQTL), population structure (genotype PCs) were adjusted for.
Population characteristics	The mean maternal age, pre-pregnancy BMI, and gestational duration was 27 years, 24.5 kg/m2, and 39 weeks, respectively. Other characteristics disaggregated by sex in the studied cohorts has been described in Supplementary Table 1.
Recruitment	The NICHD Fetal Growth Studies samples were recruited from 12 clinical sites in the US. Women with low-risk for adverse pregnancy complications were recruited from four race/ethnic groups to develop a fetal growth chart. Exclusions were applied to women with autoimmune diseases, chronic hypertension, diabetes, chronic renal disease, cancer, HIV/AIDS, or psychiatric disorders. The distribution of birthweight, placental DNA methylation and gene expression in the cohort may reflect this low-risk cohort selection. The allele frequency distribution of common SNPs may vary by ancestry, which is heterogeneous in the cohort, but adjustment for genotype principal components and inclusion of SNPs common across the entire dataset is likely to minimize ancestry-related differences, which have also been shown by other studies to be minimal in eQTL.
Ethics oversight	The NICHD Fetal Growth study protocol was approved by the institutional review boards of NICHD and each of the participating clinic sites, namely, Columbia University, New York; New York Hospital, Queens, New York; Christiana Care Health System, Delaware; Saint Peter's University Hospital, New Jersey; Medical University of South Carolina, South Carolina; University of Alabama, Alabama; Northwestern University, Illinois; Long Beach Memorial Medical Center, California; University of California, Irvine, California; Fountain Valley Hospital, California; Women and Infants Hospital of Rhode Island, Rhode Island; and Tufts University, Massachusetts. Written informed consent was obtained from all study participants.<br><br>Institutional Review Boards of Emory University and Women and Infants Hospital of Rhode Island approved the RICHS study.

Note that full information on the approval of the study protocol must also be provided in the manuscript.

Field-specific reporting

Please select the one below that is the best fit for your research. If you are not sure, read the appropriate sections before making your selection.

☒ Life sciences ☐ Behavioural & social sciences ☐ Ecological, evolutionary & environmental sciences

For a reference copy of the document with all sections, see nature.com/documents/nr-reporting-summary-flat.pdf

Life sciences study design

All studies must disclose on these points even when the disclosure is negative.

Sample size	The sample sizes for both the discovery and replication datasets were larger than recommended samples by GTEx and in most QTL studies in humans, adequately identifying mQTLs and eQTLs in cis.
Data exclusions	In the NICHD Fetal Growth Studies, low-risk pregnant women were enrolled. Exclusions included pregnant women who reported having major medical condition (cancer, autoimmune disease, diabetes, HIV or AIDS, chronic renal disease and psychiatric disorder. Prior to analysis, samples were excluded based on a pre-established quality control implemented on the genetic and molecular data to minimize bias introduced by technical artifacts. Exclusions include: discrepancies between phenotypic sex and genotypic sex, outliers from the distribution of the samples' genetic clusters based on multi-dimensional scaling plots, and a mismatching sample identifier.
Replication	Reproducibility was assessed through interrogation of published studies and using an independent dataset. Analyses identified previously known as as well as novel associations, reassuring validity. Clustering of associations in relevant biological processes ruled out chance. Analysis using an independent dataset has validated some findings of the discovery dataset. Some findings were not validated either because they were not found in the analytic dataset of the replication cohort or did not pass statistical test, potentially due to cohort-related differences such as environmental exposures, ancestry, and study design (the replication cohort being enriched for small- or large-for-gestational-age infants, whereas the discovery was largely low-risk pregnancy with birth size aligned to the appropriate-for-gestational-age distribution).
Randomization	Following the baseline interview, women were randomized to 1 of 4 ultrasonography schedules. By design, this mixed longitudinal randomization scheme captured weekly fetal growth data without exposing women to weekly ultrasound examinations. All women received upto five ultrasonography schedules throughout pregnancy, hence the randomization is unlikely to result in differences in the primary outcome (fetal growth).
Blinding	This is an observational study, hence no blinding was needed.

Reporting for specific materials, systems and methods

We require information from authors about some types of materials, experimental systems and methods used in many studies. Here, indicate whether each material, system or method listed is relevant to your study. If you are not sure if a list item applies to your research, read the appropriate section before selecting a response.

Materials & experimental systems

n/a	Involved in the study	
☒	☐	Antibodies
☒	☐	Eukaryotic cell lines
☒	☐	Palaeontology and archaeology
☒	☐	Animals and other organisms
☐	☒	Clinical data
☒	☐	Dual use research of concern
☒	☐	Plants

Methods

n/a	Involved in the study	
☒	☐	ChIP-seq
☒	☐	Flow cytometry
☒	☐	MRI-based neuroimaging

Clinical data

Policy information about clinical studies

All manuscripts should comply with the ICMJE guidelines for publication of clinical research and a completed CONSORT checklist must be included with all submissions.

Clinical trial registration	ClinicalTrials.gov, NCT00912132
Study protocol	https://clinicaltrials.gov/ct2/show/NCT00912132
Data collection	Data collection of the NICHD Fetal Growth Studies Singletons was done from July 2009 to January 2013. Singleton pregnant women were recruited at 12 participating US clinical sites and followed through pregnancy: Columbia University (NY), New York Hospital, Queens (NY), Christiana Care Health System (DE), Saint Peter's University Hospital (NJ), Medical University of South Carolina (SC), University of Alabama (AL), Northwestern University (IL), Long Beach Memorial Medical Center (CA), University of California, Irvine (CA), Fountain Valley Hospital (CA), Women and Infants Hospital of Rhode Island (RI) and Tufts University (MA).
Outcomes	Primary outcome in NICHD Fetal Growth Studies was fetal growth trajectory, create an individualized standard for fetal growth, and improve accuracy of fetal growth estimation. Fetal growth trajectories were created using 2-D ultrasound fetal biometry. Secondary outcomes include: constructing standards for fundal height, gestational diabetes mellitus (GDM) measured using clinical protocol and its association with fetal growth, impact of maternal obesity on fetal growth, collecting placental tissues and cord blood to study intrauterine growth restriction, collecting dietary intake data to study the association between maternal nutrition and fetal growth (as described in https://clinicaltrials.gov/ct2/show/NCT00912132)

Plants

Seed stocks	Not applicable
Novel plant genotypes	Not applicable
Authentication	Not applicable
